# Incidence trends of pediatric onset inflammatory bowel disease in the years 2000–2009 in Saxony, Germany–first results of the Saxon Pediatric IBD Registry

**DOI:** 10.1371/journal.pone.0243774

**Published:** 2021-01-04

**Authors:** Ivana Kern, Olaf Schoffer, Wieland Kiess, Jobst Henker, Martin W. Laaß, Ulf Winkler, Jürgen Quietzsch, Olaf Wenzel, Marlen Zurek, Katrin Büttner, Peter Fischer, Jan de Laffolie, Ulf Manuwald, Thoralf Stange, Ronny Zenker, Jens Weidner, Klaus-Peter Zimmer, Hildebrand Kunath, Joachim Kugler, Thomas Richter, Ulrike Rothe

**Affiliations:** 1 Department of Health Sciences/Public Health, Institute and Policlinic for Occupational and Social Medicine, Faculty of Medicine “Carl Gustav Carus”, TU Dresden, Dresden, Germany; 2 Center for Evidence-Based Healthcare, University Hospital and Faculty of Medicine “Carl Gustav Carus”, TU Dresden, Dresden, Germany; 3 Department of Women and Child Health, Hospital for Children and Adolescents, Center for Pediatric Research, University of Leipzig, Leipzig, Germany; 4 Children's Center Dresden-Friedrichstadt, Dresden, Germany; 5 Faculty of Medicine “Carl Gustav Carus”, University Hospital for Children and Adolescents, TU Dresden, Dresden, Germany; 6 Clinic for Children and Adolescents, Hospital Bautzen, Oberlausitz-Hospitals, Bautzen, Germany; 7 Clinic for Children and Adolescents, DRK Hospital Lichtenstein, Lichtenstein, Germany; 8 Clinic for Children and Adolescents, Helios Hospital Aue, Aue, Germany; 9 Clinic for Children and Adolescents, Hospital St. Georg, Leipzig, Germany; 10 Medical Care Centre—Polyclinic Spremberg, Spremberg, Germany; 11 General Pediatrics for Children and Adolescents, Naunhof, Germany; 12 Department of General Pediatrics, Children's Gastroenterology/Hepatology/Nutrition, Justus-Liebig-University Gießen, CEDATA-GPGE Working Group, Gießen, Germany; 13 Institute for Medical Informatics and Biometry, Faculty of Medicine “Carl Gustav Carus”, TU Dresden, Dresden, Germany; 14 Department of General Practice, Medical Clinic 3, Faculty of Medicine “Carl Gustav Carus”, TU Dresden, Dresden, Germany; 15 Faculty of Medicine “Carl Gustav Carus“, TU Dresden, Dresden, Germany; Center for Primary Care and Public Health, SWITZERLAND

## Abstract

**Aims:**

In developed countries, the incidence of inflammatory bowel disease (IBD) such as Crohn's disease (CD) and ulcerative colitis (UC) is increasing. Therefore, we aimed to investigate the incidence rates and trends over time in the population of children and adolescents in one of the federal states of Germany, in Saxony.

**Methods:**

Over the 10-year period 2000–2009 all 31 children’s hospitals and pediatric gastroenterologists, respectively in Saxony reported all IBD patients up to 15 years of age to the Saxon Pediatric IBD Registry. The completeness of the registry was estimated as 96.7% by independent surveys in the years 2005–2009. Incidence rates were presented as age-standardized incidence rates (ASR) regarding New European Standard Population 1990 per 100,000 person-years (PY) with 95% confidence intervals [CI]. Joinpoint and linear regression was used for trend analyses.

**Results:**

344 patients with confirmed IBD between 2000–2009 were included in the epidemiological evaluation: 212 (61.6%) patients with CD, 122 (35.6%) with UC and 10 (2.9%) with unclassified IBD (IBD-U). The ASR per 100,000 PY over the whole observation period was 7.2 [6.4–7.9] for IBD, 4.4 [3.8–5.0] for CD, 2.6 [2.1–3.0] for UC and 0.2 [0.1–0.3] for IBD-U. For IBD, the ASR per 100,000 PY increased from 4.6 [2.8–6.3] in 2000 to 10.5 [7.5–13.6] in 2009. The incidence trend analysis of ASRs using the joinpoint regression confirmed a significant increase of IBD as well as UC. The mean age at first diagnosis decreased significantly during the observation period from 11.5 (11.0–13.4) in 2000 to 9.6 (5.1–13.5) years in 2009. The median of the diagnostic latency among IBD patients was 3 months.

**Conclusion:**

The incidence of IBD in children and adolescents in Saxony was slightly higher than the average of other countries in the same time period and followed the trend towards a general increase of IBD. The age at diagnosis was subject to a very unfavorable downward trend.

## Introduction

Inflammatory bowel disease (IBD) mainly represented through Crohn's disease (CD) [1] and ulcerative colitis (UC) [2], constitutes an increasing problem in pediatrics [3, 4]. Causes for the increasing incidence of IBD are not clear. Factors such as nutrition (including breast-feeding), life style (e.g. stress, smoking), intestinal microflora and genetic predisposition [5–9] as well as environmental risk factors [10] may play a role in the pathogenesis of IBD. Though most patients are diagnosed with IBD in their 3^rd^ decade of life, 20% to 25% of patients are diagnosed before the age of 18 [11–13]. Incidence rates were relatively high in North America: in the USA a rate of 7.0 per 100,000 [14] was observed in 2000–2001 for children and adolescents under 18 years of age. In most Western European countries (i.e. UK, Ireland, Southern Norway, France and Scandinavia), the incidence rates per 100,000 were generally lower until 2000, and ranged between 2.5 and 5.2 [15–24] with few exceptions, e.g. Scotland: 6.4 [24]. Data in some countries have shown a significant long-term increase of incidence rates over time in the past. For example, incidence in Sweden increased from 4.6 in 1984–1986 to 7.0 per 100,000 per year comparing in 1993–1995 [19], values on incidence in Scotland increased from 4.5 (1990–1995) to 7.8 (2003–2008) per 100,000 person-years [25]. Recent trends indicate a change in incidence course [26, 27]. There is also geographical variation, such as a north-south gradient in Europe, similar to that observed in other autoimmune diseases, e.g. type-1-diabetes [26, 28, 29].

Clear epidemiological data on the previous incidence (before 2000) of pediatric IBD in Germany over a longer period is lacking. Since 1977, a children’s hospital in Leipzig, Saxony collected empirical data that indicated a clear increase of IBD incidence since 1992 [3]. This illustrated the need for an IBD register [30]. It remains to be seen, whether this rising trend will continue in the whole federal state of Saxony.

Consequently, the Saxon Pediatric IBD Registry was established in 2000 to gather reliable and valid epidemiological population-based data with the aim to observe the trend of IBD in Saxony, Germany [30].

## Methods

### Saxon Pediatric IBD Registry (first data source)

All 31 children’s hospitals in Saxony contributed data to the registry. Only two regional hospitals stated that they did not diagnose or treat any IBD patients. Consequently, all children and adolescents with IBD who were diagnosed and treated by specialized pediatric gastroenterologists in one of Saxon children's hospitals or at an outpatient clinic since 2000 were reported to the registry. The statewide population-based data collection was designed as an active prospective register.

To ensure the highest possible quality of the data basis and the results, only the subgroup of children and adolescents younger than 15 years was evaluated in total and in 3 subordinated age groups (AG): 0–4, 5–9 and 10–14 years. This also guarantees good comparability with studies from other countries in usual age classes. Children under the age of 15 are primarily treated by pediatricians in the German health care system [31].

Data were collected prospectively during the 10-year period between January 1, 2000 and December 31, 2009. The defined territory was the federal state of Saxony (18,415 km^2^, 4.3 Mio inhabitants), one of the German federal states. During the observation period, between 550,835 and 436,305 children younger than the age of 15 years lived in this area [32]. Treating hospitals sent completed standardized forms to the Medical Faculty of the Technical University (TU) Dresden. In 2017–2020, the data were supplemented and completed, ordered, complexly validated and evaluated for the first time.

IBD patients were defined as patients who met the endoscopic, histologic, imaging diagnostics, clinical and laboratory parameters according to the Lennard-Jones criteria [33], since 2005 the Porto criteria were used to define IBD [34]. Patients were followed up preferably at intervals of at least twice a year. The most frequent confirmed diagnosis in the most recent reports was evaluated. All IBD patients with an address registered in Saxony (according to their zip codes) were included.

After the diagnosis of IBD (CD, UC or unclassified IBD, short IBD-U) was confirmed [35], patient data were transmitted using the “Initial registration form” (S1 and S2 Appendices). This data form included characteristics, such as time of initial symptoms, character of symptoms, family history and the date of diagnosis (i.e. the date of therapy initiation). At the time of the first and every further presentation (either at the children’s hospital or at the outpatient clinic), a “Documentation form” was sent to the pediatric IBD registry. These forms recorded data about the clinical course and treatment. Biannual meetings of the clinicians treating IBD children were held to discuss patients’ data as well as the progress and the validation of the registry. These meetings had the aim to establish a unified approach with common diagnostic and treatment criteria.

The questionnaires were further developed and improved during the register period. In order to be able to process the data flow efficiently, all documents were transferred into a machine-readable layout (software instrument Teleform). The participating institutions were able to send the completed questionnaires both by post or fax, and paper documents were scanned and saved as images. A special fax number was set up on a separate fax server, and specially developed scripts enabled automatic, computer-supported data acquisition.

Once a year, the data in the database were reviewed completely. A list of all patients was sent to the hospitals to confirm the patients’ diagnoses and to submit information; hospitals were asked for a detailed patient chart review. In some cases, a follow-up phone interview with the physicians was carried out to verify the data. Patients were excluded from the analysis if a clear IBD diagnosis could not be established.

### Structured survey (second data source)

The completeness of the registry was confirmed via the capture-recapture method by means of an independent survey (second data source). This was done by asking 182 resident doctors (107 general practitioners, 56 specialists of internal medicine and 19 pediatricians) in the administrative district of Bautzen (area: 2,395.6 km^2^, inhabitants 2009: 279,109, children younger than 15 years: 30,027) [32] whether they treated pediatric IBD patients in 2008 and 2009 or not. The survey was initially started by postal service with a structured questionnaire and then continued per email, fax or phone in the years 2009–2010. In addition, 5 hospitals and 6 gastroenterological practices for adults in the city of Leipzig (Area: 297.4 km^2^, inhabitants 2009: 518,862, children younger than 15 years: 56,787) [32] were personally consulted in 2010–2012 to search for IBD patients treated in the years 2005–2009 [31].

All patient data from this second data source was collected pseudonymized (only initials were known) and was compared with the data of the Saxon Pediatric IBD Registry. The structured survey aimed at a complete acquisition of all possible pediatric IBD patients. After the IBD cases of the second data source had been collected, they were searched among the known patients in the registry.

### Data management and statistical analysis

The database contains complete data from all children's hospitals in Saxony over 10 years. Only those patients of the Saxon Pediatric IBD Registry who lived in Saxony (zip code of the place of residence), who were diagnosed with IBD in the 10 years period (01.01.2000–31.12.2009) and who were younger than 15 years at initial diagnosis were epidemiologically evaluated. The most recent confirmed diagnosis (according to the Porto criteria) [34] were validated for calculations.

Duplicate reports were searched, identified and eliminated prior to evaluation. Population data were obtained from the Statistical State Office of Saxony [32].

The New European Standard Population (ESP) (WHO 1990, www.gbe-bund.de) was used for the age-standardization of the incidence calculations. The age-standardized IBD incidence rates (ASR) per 100,000 person-years (PY) were calculated for children and adolescents younger than 15 years [36]. 95% confidence intervals [CI] are presented in brackets and were calculated using the Wald equation [37], estimated by means of the normal approximation. The IBD cases were also grouped by gender and in 3 age groups: 0–4, 5–9 and 10–14 years of age at diagnosis.

The joinpoint regression method was used to analyze the trend for the incidence rates within the 10 years period and to test the statistical significance of the results [38]. In joinpoint regression, logarithmically transformed models were calculated for the standardized incidence rates. Thus, the slope parameter can be expressed as annual percent change (APC). Trend changes over time were investigated. A linear regression over the individual data, with year of diagnosis as explanatory variable and age in years as dependent variable, was used to analyze trend regarding the age at first diagnosis over the years. To facilitate the interpretation of the estimated intercept, the year 2000 was coded as 0, the year 2001 as 1, etc. Calculations were carried out for all IBD patients together, for individual IBD disease forms (CD, UC and IBD-U) and separately by gender.

Diagnostic latency was defined as the difference between the diagnosis date and the self-reported beginning of symptoms of the disease. Only the month and year were recorded in the initial registration sheet. Thus, latency months were calculated for each patient and evaluated using median and interquartile range (IQR, given in brackets). Differences between genders with respect to diagnostic latency were investigated using the Mann-Whitney U test.

Trend and inference calculations were carried out with the Joinpoint Regression Program (Version 4.2.0.2, Statistical Research and Applications Branch, National Cancer Institute, Bethesda, Maryland, USA) and with R-Program (Version 3.4.3). The level of significance was defined as α = 0.05.

Other statistical evaluation was carried out with the statistical software IBM SPSS Statistics (Version 25, IBM Corporation, Armonk, New York, USA), tables and graphics for this publication were created in SPSS or Microsoft Excel (Microsoft Corporation, Redmond, WA).

### Compliance with ethical standards

#### Ethics statement

The Ethics Committee of the University of Leipzig (Reg. No. 033/2000) approved the registry design. All procedures have been performed in accordance with the ethical standards of institutional and national research, the Helsinki Declaration or comparable ethical standards. A data protection vote is available based on an informed consent from the parents of all individual underage participants included in the registry. All patient data were pseudonymized and handled confidentially in the database at the TU Dresden in accordance with data protection regulation of Federal Republic of Germany and Europe (see declaration of consent). A biunique identifier/patient-number was used within the whole database.

## Results

### First data source–Saxon Pediatric IBD Registry

During the observation period of the registry, 344 patients under the age of 15 years fulfilled the inclusion criteria and were included in the epidemiological evaluation. 67 patients (19.5%) received medical care in two clinics, 7 patients (2.0%) even in 3 clinics. About 80% of the patients were initially diagnosed in one of 6 specialized centers for pediatric gastroenterology (Leipzig University, St. Georg Hospital Leipzig, University Hospital TU Dresden, and the children’s hospitals of Bautzen, Plauen and Chemnitz).

A total of nearly 5,000 registration and documentation forms were considered. The number of reports per patient fluctuated between 2 and 80 46 patients (13.4%) had only 2 forms, and an average of 14 reports (median 8 reports) were received per patient. Sometimes the IBD diagnosis was changed several times between CD, UC and IBD-U, once for 10.2%, twice or more for 2.6% of the patients in the cohort. Initially unspecified IBDs (IBD-U) were often later specified to CD or UC (7.6%), while changes in the opposite direction were rare (0.9%). Diagnostic changes from CD to UC (2.0%) or vice versa (2.3%) also occurred.

### Second data source and completeness of the registry

After repeated attempts to survey of the second data source in the administrative district of Bautzen 180 out of 182 (98.9%) outpatient doctors replied, and 25 IBD cases were recorded. Two of the 25 patients were not found within the known patients of the Saxon Pediatric IBD Registry–since they were 16.1 and 18.1 years old. Only the subgroup of children under the age of 15 years at initial diagnosis were evaluated, so these 2 unknown patients have been excluded. All 23 remaining patients from the second data source were already listed in the registry. Additionally, 229 IBD patients were seen by a gastroenterologist for adults in the district of the city of Leipzig, 11 of whom were younger than 18 years and residing in the city of Leipzig [31]. One of these patients was under the age of 15 years and was not found in the registry. Therefore, the resulting ascertainment of completeness of the registry for children under 15 years of age was estimated to be 96.7%.

### Incidence rates and incidence trend analysis

Annual and total incidence rates of IBD in children and adolescents in Saxony between 2000 and 2009 are presented in Table 1. For better comparison with international studies, the data were also stratified by gender and in 3 age groups.

**Table 1:** Age-standardized incidence rates (ASR) of Inflammatory Bowel Disease (IBD), Crohn’s Disease (CD), Ulcerative Colitis (UC) and unclassified IBD (IBD-U) per 100,000 Person-Years (PY) for children and adolescents < 15 years of age at diagnosis in Saxony. Annual rates, total rates, gender-specific rates and age-specific incidence rates in age groups (AG) per one year.

	population at risk	IBD			CD			UC			IBD-U		
		n	ASR	95% CI	n	ASR	95% CI	n	ASR	95% CI	n	ASR	95% CI
**year**													
2000	550,835	30	**4.6**	[2.8–6.3]	21	**3.3**	[1.8–4.8]	9	**1.3**	[0.4–2.1]	0	**0.0**	-
2001	522,225	32	**4.7**	[3.0–6.3]	15	**2.2**	[1.1–3.3]	15	**2.2**	[1.1–3.3]	2	**0.3**	[0.0–0.7]
2002	494,070	42	**7.7**	[5.3–10.1]	27	**5.0**	[3.1–7.0]	14	**2.5**	[1.2–3.8]	1	**0.2**	[0.0–0.5]
2003	470,594	38	**7.5**	[5.1–9.9]	25	**5.0**	[3.0–6.9]	13	**2.6**	[1.2–4.0]	0	**0.0**	-
2004	451,952	43	**9.8**	[6.9–12.8]	28	**6.4**	[4.0–8.7]	15	**3.5**	[1.7–5.2]	0	**0.0**	-
2005	436,305	25	**6.3**	[3.8–8.8]	16	**4.0**	[2.0–6.0]	7	**1.8**	[0.5–3.1]	2	**0.5**	[0.0–1.3]
2006	437,421	31	**8.0**	[5.2–10.8]	19	**5.0**	[2.7–7.3]	11	**2.8**	[1.1–4.4]	1	**0.2**	[0.0–0.6]
2007	444,508	22	**5.8**	[3.3–8.2]	12	**3.2**	[1.4–5.0]	10	**2.6**	[1.0–4.2]	0	**0.0**	-
2008	454,198	35	**8.3**	[5.5–11.0]	23	**5.4**	[3.2–7.7]	11	**2.6**	[1.0–4.1]	1	**0.3**	[0.0–0.8]
2009	464,584	46	**10.5**	[7.5–13.6]	26	**6.1**	[3.7–8.4]	17	**3.8**	[2.0–5.6]	3	**0.7**	[0.0–1.5]
**gender**													
Male	2,422,432	195	**7.9**	[6.8–9.0]	124	**5.0**	[4.1–5.9]	63	**2.6**	[1.9–3.2]	8	**0.3**	[0.1–0.6]
Female	2,304,260	149	**6.4**	[5.4–7.4]	88	**3.8**	[3.0–4.6]	59	**2.5**	[1.9–3.2]	2	**0.1**	[0.0–0.2]
**age groups**													
AG 0–4	1,601,929	37	**2.3**	[2.1–2.6]	24	**1.5**	[1.3–1.7]	12	**0.8**	[0.6–0.9]	1	**0.1**	[0.0–0.1]
AG 5–9	1,422,589	64	**4.5**	[4.1–4.9]	37	**2.6**	[2.3–2.9]	26	**1.8**	[1.6–2.1]	1	**0.1**	[0.0–0.1]
AG 10–14	1,702,174	243	**14.3**	[13.7–14.9]	151	**8.9**	[8.4–9.4]	84	**4.9**	[4.6–5.3]	8	**0.5**	[0.4–0.6]
**Total**	4,726,692	344	**7.2**	[6.4–7.9]	212	**4.4**	[3.8–5.0]	122	**2.6**	[2.1–3.0]	10	**0.2**	[0.1–0.3]

Among IBD patients under 15 years of age, the largest subgroup consisted of 212 patients (61.6%) with CD (Table 1). UC was recorded for 122 patients (35.5%) and IBD-U for 10 patients (2.9%). Overall, the pediatric IBD patients were 56.7% male and 43.3% female. While the number of male and female UC patients (63 male, 59 female) was nearly equal, CD and IBD-U occurred predominantly at male patients (CD: 124 male and 88 female; IBD-U: 8 male, 2 female). Results in 3 age groups according to age at first diagnosis are: 10.8% of all pediatric IBD patients in Saxony were 0–4 years old, 18.6% were 5–9 years old and nearly 3/4 of all (70.6%) were 10–14 years old. In contrast to the gender distribution, no considerable differences were found between CD and UC in the distribution of age groups.

**Fig 1:**
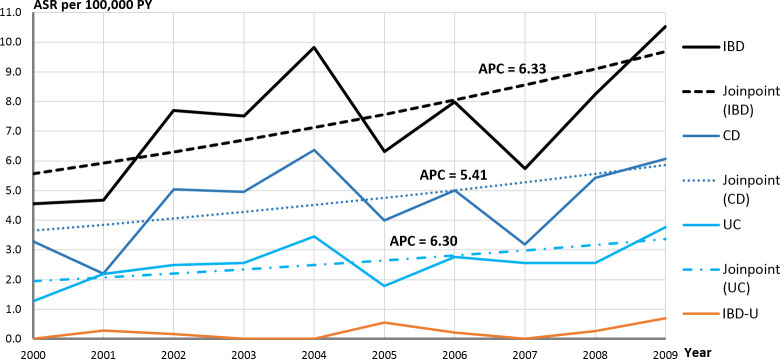
Incidence trends of age-standardized incidence rates (ASR per 100,000 PY) in Saxony, Germany by joinpoint regression with corresponding equations by all Inflammatory Bowel Disease (IBD) and separated by single diseases. Crohn’s disease (CD), ulcerative colitis (UC) and unclassified IBD (IBD-U).

In 2000, at the beginning of the observation period, the ASR per 100,000 PY was 4.6 [2.8–6.3], while at the end of this observation period in 2008 the ASR was 8.3 [5.5–11.0] and even reached 10.5 [7.5–13.6] in 2009. The observed incidence rate roughly doubled for IBD altogether, as well as for CD and UC individually (s. Fig 1).

The trend lines, which were placed over the ASR, clearly document the increase both in IBD and in all 3 diseases separately. This pattern can also be examined by gender. The rise was positive in all trend equations. A steeper slope was observed among the males than of the females. In addition, the position of the lines in the graph indicates the level of the incidence rate. The incidence of CD was higher than of UC, and the IBD incidence among males was higher than of females.

The results of the trend analysis with joinpoint regression are presented in Table 2.

**Table 2:** Results of the incidence trend analysis of Inflammatory Bowel Disease (IBD), Crohn’s Disease (CD), Ulcerative Colitis (UC) and unclassified IBD (IBD-U) for all patients and separately by gender using joinpoint regression.

disease	gender	APC %	APC 95% CI	p-value	trend
IBD	all	+ 6.3	[ 0.4–12.6]	**0.040**	significant increase
IBD	male	+ 7.3	[ 0.1–14.9]	**0.047**	significant increase
IBD	female	+ 5.5	[– 1.5–13.0]	0.108	n.s.
CD	all	+ 5.4	[– 1.9–13.3]	0.129	n.s.
CD	male	+ 5.6	[– 3.1–15.1]	0.180	n.s.
CD	female	+ 5.6	[– 4.1–16.2]	0.227	n.s.
UC	all	+ 6.3	[ 0.1–12.8]	**0.046**	significant increase
UC	male	+ 8.1	[– 1.3–18.3]	0.084	n.s.
UC	female	+ 3.1	[– 7.7–15.2]	0.538	n.s.
IBD-U	all	no estimate is possible due to zero values in the installments			

n.s. = not significant, APC = annual percent change

Significant increases of incidence over the 10 years period were ascertained for IBD- all (APC 6.3 [0.4–12.6]) and in 2 subgroups: IBD-male (APC 7.3 [0.1–14.9]) and UC-all (APC 6.3 [0.1–12.8]). For all other subgroups, positive non-significant values of APC were found. According to the joinpoint regression, no joinpoints were found for any of the groups considered. Thus, there is no evidence of a change in trends over time. The increase in ASR therefore can be regarded as continuous.

### Age-trend analysis

**Fig 2:**
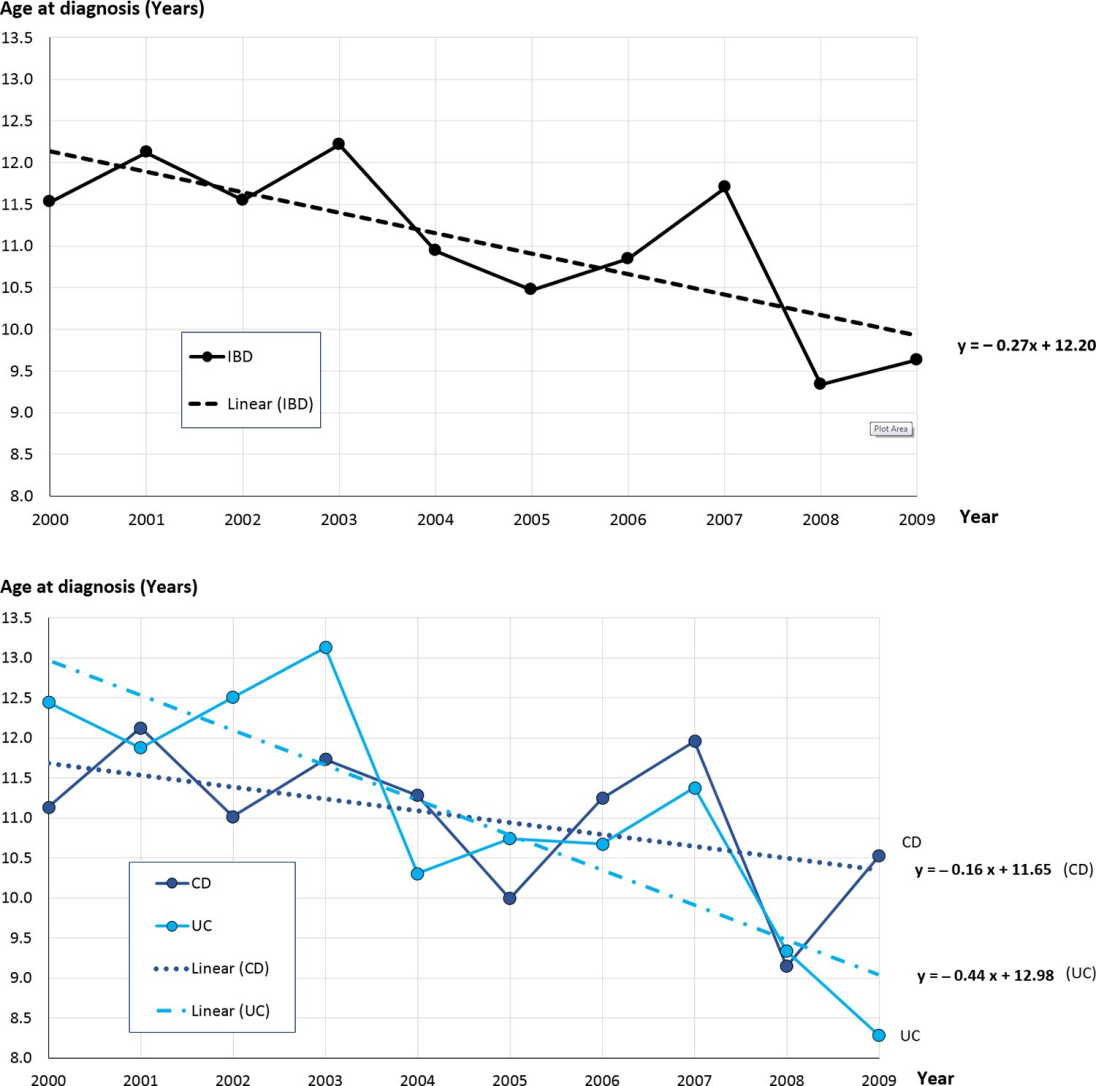
a. Age at diagnosis of patients in Saxony, Germany (mean of all cases per year) with corresponding linear trend equation by all inflammatory bowel disease patients (IBD). b. Age at diagnosis of patients in Saxony, Germany (mean of all cases per year) with corresponding linear trend equations by single diseases (Crohn’s disease (CD), ulcerative colitis (UC)).

The results and the corresponding linear trends for the age-trend analysis are shown graphically in Fig 2A and 2B.

The results for IBD-U were not presented due to the small number of cases. However, data from IBD-U patients are always included in IBD and gender-specific evaluations.

All linear trend lines y = a*x + b, that illustrate the trend under the corresponding annual means of age, had parameter a < 0, meaning age at diagnosis is decreasing. Over the observed 10 years period, children in Saxony became increasingly younger when developing IBD. This trend was observed for all IBD patients, and was approximately the same in IBD-males (a = – 0.27). Age decreased faster (a = – 0.34) for IBD-females. For single diagnoses, the age decreased faster for UC patients (a = – 0.44) than for CD patients (a = – 0.16).

In 2000, the mean age at diagnosis was 11.5 (IQR: 11.0–13.4) years, but in 2009 only 9.6 (IQR: 5.1–13.5) years. The youngest patient was 8 months old. There were only slight differences in median age when comparing by gender and by diagnosis (between CD and UC patients) as well.

Table 3 summarizes statistical test results for age trend.

**Table 3:** Results of the age trend analysis of Inflammatory Bowel Disease (IBD), Crohn’s Disease (CD), Ulcerative Colitis (UC) and unclassified IBD (IBD-U) using linear regression with trend lines y = a*x+b.

disease	parameter		std. error	t-value	p-value	trend
IBD	a =	**–** 0.27	0.07	**–** 4.089	**< 0.001**	significant decrease
IBD	b =	12.20	0.35	34.522	**< 0.001**	
CD	a =	**–** 0.16	0.09	**–** 1.801	0.073	n.s.
CD	b =	11.65	0.47	24.959	**< 0.001**	
UC	a =	**–** 0.44	0.10	**–** 4.384	**< 0.001**	significant decrease
UC	b =	12.98	0.54	23.963	**< 0.001**	
IBD-U	a =	**–** 0.47	0.44	**–** 1.066	0.318	n.s.
IBD-U	b =	14.02	2.76	5.075	**< 0.001**	

n.s. = not significant

A significant decrease of age at diagnosis were ascertained for IBD of all patients (trend line– 0.27*x + 12.20) and for UC patients (trend line– 0.44*x + 12.98), see Fig 2A and 2B. For all other subgroups it was also decreasing (a < 0), but did not reach statistical significance, for CD very close to it (and non-significant trend was observed).

### Latency of diagnosis

The general latency among all IBD patients was 3 months (IQR: 1–8 months), and this varied slightly for the different diagnoses: 4 months (IQR: 2–8) for CD, 3 months (IQR: 1–8) for UC and 4 months (IQR: 1–12) for IBD-U. For gender in age groups (s. Table 4).

**Table 4:** Latency of diagnosis (median; months) and interquartile range (IQR; months) of Inflammatory Bowel Disease (IBD) in age groups and by gender.

age groups	IBD total			IBD male			IBD female		
	cases (n)	median (months)	IQR	cases (n)	median (months)	IQR	cases (n)	median (months)	IQR
AG 0–4	37	7.0	(3.0–16.0)	23	12.0	(2.0–16.0)	14	5.5	(4.0–13.0)
AG 5–9	64	2.0	(1.0–8.0)	32	2.5	(1.0–7.0)	32	2.0	(1.0–9.0)
AG 10–14	243	3.0	(1.0–7.0)	140	4.0	(2.0–6.0)	103	3.0	(1.0–8.0)
total	344	3.0	(1.0–8.0)	195	4.0	(1.0–8.0)	149	3.0	(1.0–9.0)

**Fig 3:**
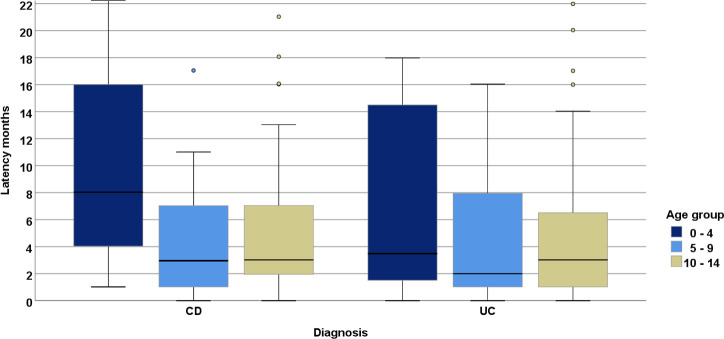
Latency months in 3 age groups using boxplots and separated by diagnosis: Crohn’s Disease (CD) and Ulcerative Colitis (UC).

Graphical representation of the latency months in age groups and by single diagnoses is presented in Fig 3.

The longest time to diagnosis was determined for youngest children aged 0–4 years, and the shortest in children aged 5–9 years. The latency among females was shorter than among males, while CD patients had generally longer latency periods than UC patients. However, gender differences were not significant (p = 0.7586). Among the oldest children (AG 10–14 years), the median value for all 3 diseases was the same (3 months) and the IQR was the lowest of all. Due to having only a few cases, IBD-U was not shown in the boxplot. Most IBD-U patients were in the 10–14 year age group, with a latency period of 3 (IQR: 1–8) months.

## Discussion

**Fig 4:**
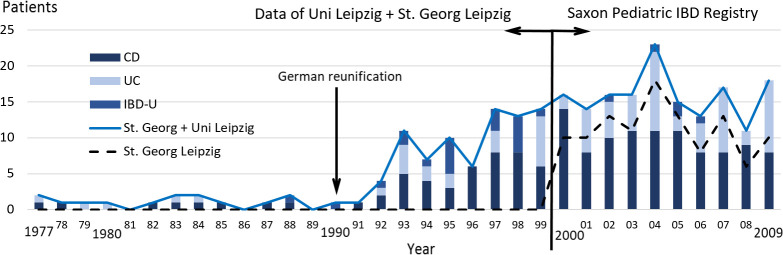
Increasing number of IBD patients in Leipzig/Saxony. Origin of the data: St. Georg Hospital, Leipzig, Germany, University Hospital for Children and Adolescents, University of Leipzig; Data till 1999 [3], since 2000 Saxon Pediatric IBD Registry.

Before 2000, reliable and population based IBD data for children and adolescents in Germany were not available. The Saxon IBD Registry made a significant contribution to the epidemiology of IBD in Germany since 2000. Our epidemiological data confirmed an increasing incidence of IBD in a German federal state, in Saxony, already suspected as a result of empirical data collected in a pediatric gastroenterology center in Saxony (Leipzig city) in 1977–1999 [3]. Thus, a unique, uninterrupted series of data over 33 years can be presented in Fig 4.

During the first 15 years observation period between 1977 and 1991 –before the establishment of the Saxon IBD registry–only 16 patients were treated with the diagnosis of IBD at the University Children’s Hospital in Leipzig (s. Fig 4). Between 1992 and 1999, already 7 to 14 patients were treated there annually [3]. But since 2000 much more patients, approximately more than 15 have been treated in Leipzig annually based on the Saxon IBD registry data.

After World War II, Germany was divided into two states with different political and economic regimes. Saxony was a part of the socialist German Democratic Republic (GDR) until the reunification in 1990. After this the population quickly adopted the so-called Western lifestyle. Whether and to what extent the increase of IBD in children and adolescents in the early 1990s and later–and the increasing incidence of type-1-diabetes after the German reunification [28]–may be due to the modified living conditions, changes to stress levels in everyday life, altered nutrition and other environmental factors, or a result of increased awareness and advanced diagnostic methods is not known and should be considered in further studies. However, in developed countries an increase in incidence of IBD has been described in many independent studies [26, 39].

Using the Saxon Pediatric IBD Registry, we determined the total pediatric (age <15) incidence rate of IBD per 100,000 PY was 7.2 [95% CI 6.4–7.9], as well as the incidence trends during the years 2000–2009 in a German federal state with 4.3 million inhabitants. Therefore, given an equal distribution of IBD in all of Germany, a country with an average of 11.7 million inhabitants up to 14,99 years of age [32], it can be approximated that about 8,500 incident pediatric IBD cases occurred in this 10 years period. The proportion CD:UC was nearly 2:1, and was similar to other observations [40]. Thus, almost 2/3 of all IBD cases are expected to be CD.

During the 10 years period, the ASR of IBD per 100,000 PY increased significantly from 4.6 [2.8–6.3] in 2000 to 10.5 [7.5–13.6] in 2009.

Comparing the results from Saxony, Germany with other epidemiological studies in other countries, we found similar results. For example, the US study from 2000–2001 in Wisconsin reported almost the same IBD incidence rate per 100,000 of 7.0 for children under 18 years [14] as the Saxon IBD register from 2000–2009 for children under 15 years of age (ASR = 7.2/100,000 PY). Different study designs, various data acquisition and data sources makes comparisons difficult. However, most recent studies, including our registry in Saxony, have the advantage of using a prospective design. Although the incidences of pediatric IBD vary between countries, the systematic review by Benchimol et al. 2011 [39] showed a similar general upward trend in recent decades (until 2006). Most studies showed an increase of incidence: 78% of the studies with IBD, 60% with CD and 20% with UC. The review by Sýkora et al. 2018 [41] likewise described the upward incidence trend (until 2018): 67% studies with CD and 46% with UC. The results from Saxony fully confirm this pattern; continuous increasing incidence trends were found both, in the overall evaluation of IBD and in subgroups of CD und UC patients, as well in both genders. This trend was observed worldwide (also in newly industrialized countries), and in adults as well as in children [39, 42–61]. Although, the incidence in Western countries has recently begun to stabilize in the 21^st^ century [27].

The global incidence rate of pediatric CD per 100,000 in 2000–2009 ranged approximately 1.0–7.0, and for UC the rates ranged 0.5–5.0 [25, 39, 41, 53–60, 62], with the exception of some North American and European regions [50–52].

Known study results from Germany were in the middle range [63]. The rates per 100,000 PY from our population based registry in Saxony (CD: 4.4 [3.8–5.0] and UC: 2.6 [2.1–3.0]) were higher than those reported by Ott et al. 2008 for a rural region of southern Germany in 2004–2006 [63], but still in the upper average of the review results [39, 41]. However, clear epidemiological pediatric data in Germany are rare. Wittig at al. (2019) recently studied health administrative data and found incidence values among the highest in the literature [64]. However, due to its procurement design it might not be comparable. In Europe, a higher incidence was reported in Western European countries, but an increasing incidence can presently be seen in Eastern European countries [44, 65]. A similar trend has been described for other autoimmune diseases, e.g. type-1-diabetes, too [28, 66, 67].

According to the literature, up to 15–23% of the IBD cases, CD and UC could not be clearly distinguished from one another; therefore, these patients were classified with IBD-U [35, 68]. After a long-term observation and re-evaluation in Saxony only 2.9% of patients with IBD were classified with IBD-U; the corresponding relatively low incidence rate is 0.2/100,000 PY. Studies in the USA and Western Europe [13, 14, 16, 24] have determined incidence rates ranging 0.1–0.7/100,000 PY, while many other authors [15, 19, 39, 69] do not provide information on the IBD-U.

As for the onset of symptoms, the literature consistently reports that IBD are serious chronic diseases, and around 15–25% (up to 30%) of all patients are already affected in infancy and adolescence [70, 71], or before the age of 18 [11–13]. In principle, the first manifestation can already occur at any age from early childhood [63, 72]. Almost 11% of the children in the Saxon registry were 4 years old or younger; almost 30% were younger than 10 years. However, there is a disagreement about the highest peak in age-specific incidence [3, 12, 21, 68] depending on the completeness of data or due to different methodological aspects. Many studies reported that patients’ onset was in their 3rd decade of life [11–13].

The mean of age at diagnosis for all pediatric IBD patients and in all subgroups (except IBD-U) was about 11 years in Saxony. Due to the concerns about the completeness and the quality of the data, older adolescents (15–18) were not evaluated. However, it was found that the number afflicted with IBD could be enormous among older adolescents [31].

A very worrying fact that has been identified in Saxony is the trend towards younger pediatric IBD patients being diagnosed. This has been shown for both diseases (CD and UC) and for both genders. A significant decrease was determined for IBD and UC, for CD the decrease was not statistically significant. Moreover, this decline in age is also observed in type-1-diabetic patients [28, 66]. Statements concerning age at diagnosis of IBD are inconsistent in the literature. In Canada the incidence has been rising most rapidly in children under 5 years of age within the past two decades [73]. In Switzerland a shift towards the IBD occurring at a younger ages has been not confirmed since 1980 [74].

The latency period between symptom onset until diagnosis was longer for CD than for UC (median 4 vs. 3 months), and was similar to that observed from the CEDATA database (median 0.5 vs. 0.3 years) [4]. For all IBD patients we observed a latency period of 3 months (IQR 1–8), which is similar to the 4 months (2–8) reported elsewhere [75]. Warning symptoms associated with UC, such as visible fecal blood, may lead parents to seek medical help for their children more urgently. In all 3 age groups, the analogous difference in latency between genders (male > female, not statistically significant) is very interesting. Maybe, female patients of all ages are able to communicate their illness better.

### Strengths and limitations

The results rest upon over 10 years of population-based registry data with excellent quality from all over Saxony. The registry has an estimated ascertainment of completeness of 96.7% for children younger than 15 years (nearly full census). Due to this fact, the incidence trend of pediatric IBD in the state of Saxony has been reliably determined.

In Germany, children and adolescents up to the age of 18 years are usually cared for and treated by pediatricians both on an outpatient and inpatient basis. Nevertheless, in adolescents older than 15 years of age, it is possible that an adult gastroenterologist has cared for them [31]. With the aim of guaranteeing the highest data quality, only age groups up to 14.99 years were evaluated epidemiologically in our study.

In order to promote participation in the registry and to promote the aim of complete data entry, semi-annual meetings were organized by the broad working group of registry members (s. acknowledgement). Procedures, diagnostics and therapies could be discussed, optimized and unified at these meetings. An internal data validity control within the database and an external re-assessment were carried out regularly.

The challenge of differentiating between individual diagnoses is mastered more reliably with the length of treatment. The relatively long duration of the Saxon registry with the small number of final IBD-U cases (2.9%) confirms the outstanding diagnostic certainty of the study.

The problem of appropriate diagnosis (classification and evaluation of the patient in the correct diagnosis group) was given the highest attention. The evaluation of the registry benefits in most cases from years of patient follow-up and ultimately also from the time interval between publication and the survey period–the query in 2018 was a significant step towards further validation. The first diagnosis made on the “Initial registration form” was not evaluated, rather the most frequent diagnosis in the most recent reports, which logically contains more information input.

Our study is subject to a causal, logical dialectic. The low number of IBD-U diagnoses is very positive, as a result of a generally mature diagnostic procedure. Nevertheless, all single IBD-U sub-results cannot represent any significant scientific weight due to the small number of cases and were only listed for documentation.

There was variation in the number of presentations to the doctor reported–the information level per IBD case differed, and the number of reports per patient fluctuated. This may have played only a minor role in the quality of the epidemiological evaluations presented here, including all considerations regarding the exact diagnosis. All IBD-U cases were clarified as best as possible and the mutual change of diagnosis from CD to UC or vice versa was observed to be about the same in the registry.

## Conclusions

The Saxon Pediatric IBD Registry presents important trends–the significant increase of incidence rates over time and a decrease in age at diagnosis–based on complete and valid, population-based data of an entire federal state of Germany over a period of 10 years. Further monitoring of the development in Saxony and comparison with other high-quality epidemiological data will be very worthwhile.

## Supporting information

S1 Appendix Questionnaire initial registration form–original in German. (PDF)

S2 Appendix Questionnaire initial registration form–translation in English. (PDF)
