# Supplementary material for: Incidence trends of pediatric onset inflammatory bowel disease in the years 2000–2009 in Saxony, Germany–first results of the Saxon Pediatric IBD Registry
Source: PLoS One. 2021 Jan 4;16(1):e0243774. doi: 10.1371/journal.pone.0243774 (PMC7781364; doi:10.1371/journal.pone.0243774)
Supplement: S1 Appendix — (PDF) [file pone.0243774.s001.pdf]

Name, Vorname des Versicherten geb. am
Kassen-Nr. | Versicherten-Nr. | Status
Betriebsstätten-Nr. | Arzt-Nr. | Datum

Kinder-CED-Register - Sachsen -

CHRONISCH ENTZÜNDLICHE DARMERKRANKUNGEN (CED) BEI KINDERN UND JUGENDLICHEN

ERSTMELDEBOGEN

Ambulanz: ☐☐☐

Vorstellungsdatum: ☐☐☐☐☐☐☐☐

Einverständniserklärung: ○ ja ○ nein Geburtsdatum Kind: ☐☐☐☐☐☐☐☐

Geschlecht: ○ männlich ○ weiblich Postleitzahl (PLZ): ☐☐☐☐☐

Geburtsdatum Mutter: ☐☐☐☐☐☐☐☐ Größe: (Mutter) ☐☐☐,☐ cm Größe: (Vater) ☐☐☐,☐ cm

Diagnose

○ **Morbus Crohn** ○ **Colitis ulcerosa** ○ **Colitis indeterminata**

Anamnese

Gewicht: ☐☐☐,☐ kg Größe: ☐☐☐,☐ cm (bei Diagnosestellung, sofern vorhanden)

Auftreten erster Symptome: ☐☐ / ☐☐☐☐ (Monat / Jahr)

Symptomatik bis zur Diagnosestellung:

- ☐ sichtbares Blut im Stuhl
- ☐ Durchfall
- ☐ Bauchschmerzen
- ☐ Gewichtsstillstand/-verlust
- ☐ Wachstumsstörung
- ☐ Fieber
- ☐ Anämie
- ☐ Leistungsknick
- ☐ Perianalfistel
- ☐ sonstige Fistel
- ☐ Perianale Läsion
- ☐ Appetitlosigkeit
- ☐ andere: ____________________

Extraintestinale Symptome: ○ **keine**

- ☐ Auge ☐ Fieber > 38,5°C > 3 Tage
- ☐ Haut
- ☐ Leber/Gallenwege/Pankreas, insbesondere ☐ PSC
- ☐ Gelenk: Entzündung ☐ Gelenke: Schmerzen
- ☐ Wirbelsäule
- ☐ andere: ____________________

Datum der Diagnosestellung: ☐☐ / ☐☐☐☐ (Monat / Jahr)

Diagnosestellung durch:
- ○ Kindergastroenterologe
- ○ anderer Kinderarzt
- ○ Gastroenterologe, Erwachsene
- ○ Internist / Allgemeinmediziner
- ○ Kinderchirurg / Chirurg

Diagnosestellung in:
- ○ eigener Klinik
- ○ Praxis
- ○ anderer Klinik ____________________

Anzahl leiblicher Geschwister: Schwestern: ☐☐ Brüder: ☐☐ Zwillingsgeschwister? ☐

CED in der Familie: (nur leibliche!) ○ nein

	Mutter	Vater	Geschwister	2. Grades
Morbus Crohn	☐	☐	☐☐	☐
Colitis ulcerosa	☐	☐	☐☐	☐

(Version 06/18)

Ausfüll-Datum: **Unterschrift:** ☐
