# Supplementary material for: Incidence trends of pediatric onset inflammatory bowel disease in the years 2000–2009 in Saxony, Germany–first results of the Saxon Pediatric IBD Registry
Source: PLoS One. 2021 Jan 4;16(1):e0243774. doi: 10.1371/journal.pone.0243774 (PMC7781364; doi:10.1371/journal.pone.0243774)
Supplement: S2 Appendix — (PDF) [file pone.0243774.s002.pdf]

Automated,
individual related data
of the health insurance

INFLAMMATORY BOWEL DISEASE (IBD) IN CHILDREN AND ADOLESCENTS

INITIAL REGISTRATION FORM

Pediatric IBD Registry - Saxony -

Outpatient department: ☐☐☐

Date of visit: ☐☐☐☐☐☐☐☐
Presentation of the patient

Informed consent: ○ yes ○ no   Date of birth - Child: ☐☐☐☐☐☐☐☐

Gender: ○ male ○ female   Zip Code: ☐☐☐☐☐

Date of birth - Mother: ☐☐☐☐☐☐☐☐   Height: ☐☐☐,☐ cm (Mother)   Height: ☐☐☐,☐ cm (Father)

Diagnosis

○ **Crohn`s disease**   ○ **Ulcerative colitis**   ○ **Unclassified IBD**

Medical History

Weight: ☐☐☐,☐ kg   Height: ☐☐☐,☐ cm   (At the date of the diagnosis, if available)

Occurrence of first symptoms: ☐☐ / ☐☐☐☐ (Month/Year)

Symptomatology until diagnosis:

- ☐ Visible fecal blood
- ☐ Diarrhea
- ☐ Abdominal aches
- ☐ Weight loss/stagnation
- ☐ Growth retardation
- ☐ Pyrexia
- ☐ Anemia
- ☐ Poor performance
- ☐ Perianal fistula
- ☐ Other fistulae
- ☐ Perianal lesion
- ☐ Loss of appetite
- ☐ Other: ______

Extraintestinal symptoms: ○ **None**

- ☐ Eyes
- ☐ Pyrexia >38.5°C > 3 days
- ☐ Skin
- ☐ Liver/Bile tracts/ Pancreas, especially ☐ PSC
- ☐ Joints: Inflammation
- ☐ Joints: Pains
- ☐ Spinal column
- ☐ Other: ______

Date of Diagnosis: ☐☐ / ☐☐☐☐ (Month/Year)

Diagnosis by:
○ **Pediatric gastroenterologist**
○ **Another pediatrician**
○ **Gastroenterologist for adults**
○ **Internist / general practitioner**
○ **Pediatric surgeon / Surgeon**

Diagnosis at:
○ **Own clinic**
○ **Practice**
○ **Another clinic** ______

Number of biological siblings: Sisters: ☐☐ Brothers: ☐☐ Twins? ☐

IBD in the family: ○ no
(only biological relatives!)

	Mother	Father	Siblings	2nd Grade
Crohn`s disease	☐	☐	☐☐	☐
Ulcerative colitis	☐	☐	☐☐	☐

(Version 06/18)

Date of Completion: **Signature:** ☐
